# Knowledge and training in paediatric medical traumatic stress and trauma-informed care among emergency medical professionals in low- and middle-income countries

**DOI:** 10.1080/20008198.2018.1468703

**Published:** 2018-05-08

**Authors:** Claire Hoysted, Franz E. Babl, Nancy Kassam-Adams, Markus A. Landolt, Laura Jobson, Claire Van Der Westhuizen, Sarah Curtis, Anupam B. Kharbanda, Mark D. Lyttle, Niccolò Parri, Rachel Stanley, Eva Alisic

**Affiliations:** a School of Psychological Sciences and Monash Institute of Cognitive and Clinical Neuroscience, Monash University, Melbourne, Australia; b Emergency Department, Royal Children’s Hospital, Melbourne, Australia; c Emergency Research, Murdoch Children’s Research Institute, Melbourne, Australia; d Department of Paediatrics, University of Melbourne, Melbourne, Australia, on behalf of the Paediatric Research in Emergency Departments International Collaborative (PREDICT) and the Pediatric Emergency Research Networks (PERN); e Centre for Injury Research and Prevention, Children’s Hospital of Philadelphia, Philadelphia, PA, USA; f Department of Pediatrics, University of Pennsylvania, Philadelphia, PA, USA; g Department of Psychosomatics and Psychiatry, University Children’s Hospital Zurich, Zurich, Switzerland; h Division of Child and Adolescent Health Psychology, Department of Psychology, University of Zurich, Zurich, Switzerland; i Department of Psychiatry and Mental Health University of Cape Town, Alan J. Flisher Centre for Public Mental Health, Cape Town, South Africa; j Departments of Pediatrics & Emergency Medicine & Women and Children’s Health Research Institute, University of Alberta, Edmonton, Canada, on behalf of the Pediatric Emergency Research Canada (PERC); k Department of Pediatric Emergency Medicine, Children’s Hospitals and Clinics of Minnesota, Minneapolis, USA, on behalf of the Pediatric Emergency Medicine Collaborative Research Committee of the American Academy of Pediatrics (PEMCRC); l Emergency Department, Bristol Royal Hospital for Children, Upper Maudlin Street, Bristol, UK, on behalf of the Paediatric Emergency Research in the UK and Ireland (PERUKI); m Faculty of Health and Applied Sciences, University of the West of England, Bristol, UK; n Department of Emergency Medicine and Trauma Center, Meyer University Children’s Hospital, Florence, Italy, on behalf of the Research in European Pediatric Emergency Medicine (REPEM); o Department of Emergency Medicine, University of Michigan, Ann Arbor, USA, on behalf of the Pediatric Emergency Care Applied Research Network (PECARN); p Monash University Accident Research Centre, Monash University, Melbourne, Australia

**Keywords:** Paediatric injury, child traumatic stress, psychological first aid, psychosocial care, traumatic stress, lesión pediátrica, estrés traumático infantil, primeros auxilios psicológicos, cuidado psicosocial, estrés traumático, 小儿损伤, 儿童创伤性应激, 心理急诊, 心理社会护理, 创伤性应激, • Emergency staff in low- and middle-income countries (LMICs) showed knowledge gaps with regard to paediatric medical traumatic stress associated with childhood injury.• Knowledge of paediatric medical traumatic stress in injured children was associated with having had training in psychosocial care and working in a higher income country within LMICs.• Emergency staff in LMICs demonstrated a need and desire for education on paediatric medical traumatic stress in injured children and training in trauma-informed care, with the majority preferring training be delivered online.

## Abstract

**Background**: Provision of psychosocial care, in particular trauma-informed care, in the immediate aftermath of paediatric injury is a recommended strategy to minimize the risk of paediatric medical traumatic stress.

**Objective**: To examine the knowledge of paediatric medical traumatic stress and perspectives on providing trauma-informed care among emergency staff working in low- and middle-income countries (LMICs).

**Method**: Training status, knowledge of paediatric medical traumatic stress, attitudes towards incorporating psychosocial care and barriers experienced were assessed using an online self-report questionnaire. Respondents included 320 emergency staff from 58 LMICs. Data analyses included descriptive statistics, *t*-tests and multiple regression.

**Results**: Participating emergency staff working in LMICs had a low level of knowledge of paediatric medical traumatic stress. Ninety-one percent of respondents had not received any training or education in paediatric medical traumatic stress, or trauma-informed care for injured children, while 94% of respondents indicated they wanted training in this area.

**Conclusions**: There appears to be a need for training and education of emergency staff in LMICs regarding paediatric medical traumatic stress and trauma-informed care, in particular among staff working in comparatively lower income countries.

## Abstract (es)

**Introducción**: Proveer atención psicosocial, en particular atención informada sobre el trauma, inmediatamente después de una lesión pediátrica es una estrategia recomendada para minimizar el riesgo de estrés traumático en medicina pediátrica. Objetivo: Examinar el conocimiento del estrés traumático en medicina pediátrica y las perspectivas para proporcionar atención informada sobre el trauma entre el personal de emergencias que trabaja en países de ingresos bajos y medios (PIBM).

**Método**: Se evaluó el estado de capacitación, el conocimiento del estrés traumático en medicina pediátrica, las actitudes sobre la incorporación de la atención psicosocial y las barreras experimentadas mediante un cuestionario de autoinforme en línea. Los participantes fueron 320 individuos que pertenecían al personal de emergencia de 58 PIBM. Los análisis de datos incluyeron estadísticas descriptivas, pruebas t y regresión múltiple.

**Resultados**: El personal de emergencia que trabajaba en PIBM tenía un nivel bajo de conocimiento del estrés traumático en medicina pediátrica. El noventa y uno por ciento de los encuestados no había recibido ningún entrenamiento o educación en el estrés traumático en medicina pediátrica, o en atención informada en trauma para niños con lesiones, mientras que el 94% de los encuestados indicó que quería capacitación en esta área.

**Conclusiones**: Parece que es necesario capacitar y educar al personal de emergencia en PIBM en relación al estrés traumático en medicina pediátrica y sobre la atención informada en trauma, en particular entre el personal que trabaja en países de ingresos relativamente bajos.

## Abstract (zh)

背景：将儿科医疗创伤性应激风险降至最低的推荐策略是，在儿童伤害后立即提供社会心理护理，特别是创伤知情护理。

目的：考察儿科医学创伤性应激的知识以及在低收入和中等收入国家（LMICs）工作的急诊人员中提供创伤护理的观点。

方法：使用在线自我报告调查问卷评估培训状况、儿童医学创伤应激知识，对纳入社会心理护理的态度和经历的障碍。 受访者包括来自58个LMIC的320名急诊人员。数据分析包括描述性统计，t检验和多元回归。

结果：在中低收入国家工作的紧急诊援人员对儿科医学创伤性压力知识水平偏低。 91％的受访者没有接受任何儿科医学创伤性应激的培训或教育，或受伤儿童的创伤性知识护理，而94％的受访者表示他们想要在这方面进行培训。

结论：似乎有必要对中低收入国家的急诊医务人员进行培训和教育，特别是在收入相对较低的国家的工作人员，这些工作涉及儿童医疗创伤性应激和创伤护理。

Internationally, millions of children suffer acute injuries each year, with the global burden of childhood injuries being disproportionately higher in low- and middle-income countries (LMIC) than in high-income countries (Ameratunga, Hijar, & Norton, 2006; Chandran, Hyder, & Peek-Asa, 2010; Peden et al., 2008; World Health Organization, 2016). Childhood injuries can have profound and lifelong psychological effects, with approximately one in six injured children experiencing psychological sequelae, including paediatric medical traumatic stress (Kassam-Adams, Marsac, Hildenbrand, & Winston, 2013; Price, Kassam-Adams, Alderfer, Christofferson, & Kazak, 2015). Paediatric medical traumatic stress is defined as ‘a set of psychological and physiological responses of children and their families to pain, injury, serious illness, medical procedures and invasive or frightening treatment experiences’ (National Child Traumatic Stress Network, 2003). Paediatric medical traumatic stress can impair typical childhood development, result in lower health-related quality of life, compromise physical functioning and recovery, and lead to a greater reliance on the health care system (Landolt, Vollrath, Gnehm, & Sennhauser, 2009; Marsac, Kassam-Adams, Delahanty, Widaman, & Barakat, 2014; Price et al., 2015).

Posttraumatic stress symptoms, such as those evident in paediatric medical traumatic stress, have been demonstrated to exist across cultures and societies (Dyregrov, Gupta, Gjestad, & Raundalen, 2002; Ehntholt & Yule, 2006, p. 1198; Foa, Keane, Friedman, & Cohen, 2008); with studies of children and adolescents from LMICs including Afghanistan (Mghir, Freed, Raskin, & Katon, 1995), Bosnia-Hercegovina (Papageorgiou et al., 2000; Smith, Perrin, Yule, Hacam, & Stuvland, 2002), Cambodia (Sack, Seeley, & Clarke, 1997), India (Kar et al., 2007), Kuwait (Nader, Pynoos, Fairbanks, Al-Ajeel, & Al-Asfour, 1993), Lebanon (Saigh, 1991), Palestine (Thabet & Vostanis, 1999) and Rwanda (Dyregrov, Gupta, Gjestad, & Mukanoheli, 2000). Further, there is cumulating evidence demonstrating that risk factors for the development of posttraumatic stress symptoms in children, adolescents and adults are consistent across cultures (Dyregrov et al., 2002; Ehntholt & Yule, 2006; Sack et al., 1997; Schnyder et al., 2016; Smith et al., 2002). Despite evidence that traumatic stress impacts children in LMICs (which are burdened with high rates of injuries and childhood trauma exposure) there is limited systematic research conducted in LMIC settings examining the efficacy of treatment approaches (Chandran et al., 2010; Foa et al., 2008; Fodor et al., 2014). Furthermore, many LMICs have limited access to resources, training and education and poor health infrastructure, which impacts implementation of evidence-based treatments and preventative measures (Chandran et al., 2010; Schnyder et al., 2016).

Research has highlighted the need for preventative approaches in reducing the risk of the development of paediatric medical traumatic stress (Horowitz, Kassam-Adams, & Bergstein, 2001; Kassam-Adams, 2014; Kassam-Adams & Butler, 2017). Due to the greater prevalence of injury and trauma, and resource limitations in LMICs, particular emphasis has been placed on addressing this need in LMICs (Ameratunga et al., 2006; Gunaratnam & Alisic, 2017). The peri-traumatic period immediately post-injury has been identified as a critical period for the prevention of the development of paediatric medical traumatic stress. It is in this period that interventions can still target the child’s subjective experience of the event (Kazak et al., 2006; Marsac et al., 2014). Research has recognized the important role that medical and nursing professionals, who treat injured children in this acute peri-traumatic period, have in the prevention and identification of paediatric medical traumatic stress (Horowitz et al., 2001; Kassam-Adams, 2014; Marsac et al., 2015; Patel et al., 2007). In LMICs this includes medical and nursing professionals who provide emergency medical care (for simplicity henceforth emergency care nurses and physicians will be referred to as *emergency staff*). Given limited resources in some LMIC areas, this has been identified as one method for improving access to evidence-based treatments in LMICs (Patel, Chowdhary, Rahman, & Verdeli, 2011; van Ginneken et al., 2013). Such integration of mental health interventions into emergency care has been highlighted as a cost-effective and feasible method of delivering mental health prevention (Kassam-Adams, 2014; Patel et al., 2007).

One approach to prevention is the provision of psychosocial care, in particular trauma-informed care (Magruder, Kassam-Adams, Thoresen, & Olff, 2016; Marsac et al., 2015). Trauma-informed care is a type of psychosocial care that can be incorporated into standard acute medical care and involves acknowledging the prevalence of trauma; recognizing how trauma can affect everyone who experiences the potentially traumatic event (including the child, family and emergency staff); responding by incorporating an understanding of the impact of trauma into practice; and aiming to prevent further trauma (Substance Abuse and Mental Health Services Administration, 2014). This approach can be incorporated into acute medical care and when provided in the immediate aftermath of paediatric injury aims to change the subjective experience of the potentially traumatic event. This is achieved by reducing pain and distress, providing emotional support and promoting both physical and psychological recovery, thus minimizing the risk of the development of paediatric medical traumatic stress (Ko et al., 2008; Marsac, Kassam-Adams, Hildenbrand, Kohser, & Winston, 2011; Marsac et al., 2015). As such, trauma-informed care, provided by emergency staff, is indicated as a low-cost, low-intensity preventative measure for targeting paediatric medical traumatic stress in LMICs (Ko et al., 2008; Marsac et al., 2015). Despite being the recommended approach, research is needed to examine the effectiveness of trauma-informed care as a preventative approach for paediatric medical traumatic stress (De Young & Kenardy, 2017; Marsac, Hildenbrand, & Kassam-Adams, 2017; Marsac et al., 2015). Examining the effectiveness of trauma-informed care in this setting requires emergency staff to integrate this approach into their practice. However, research has consistently demonstrated that emergency staff are lacking knowledge of psychological difficulties, such as paediatric medical traumatic stress and skills in promoting psychological recovery utilizing a trauma informed care approach (Banh, Saxe, Mangione, & Horton, 2008; Hoysted et al., 2017; Kassam-Adams et al., 2015; Ziegler, Greenwald, DeGuzman, & Simon, 2005). Further, research has found a greater need for training in LMICs (Alisic et al., 2016) potentially explained by the lack of available resources, including professional training and education, in many LMICs (Chandran et al., 2010; Hsia, Thind, Zakariah, Hicks, & Mock, 2015; Obermeyer et al., 2015). Therefore, the development of targeted training programmes is required to address these limitations in emergency staff knowledge in these economic regions.

The aim of the current study was to conduct further analysis to explore LMIC emergency staff’s knowledge of paediatric medical traumatic stress and perspectives on providing psychosocial care in order to inform the development of training programmes as a first step towards addressing the gaps identified in the literature. In particular, we aimed to understand: (1) the proportion of emergency staff in trained in any form of psychosocial care for injured children and their preferences for further training; (2) emergency staff’s knowledge of paediatric medical traumatic stress in children; (3) what barriers to implementing trauma-informed care are experienced by emergency staff; and (4) emergency staff’s attitudes to providing trauma-informed care. It is hypothesized that emergency staff in LMICs will demonstrate a need for training, have a positive attitude towards providing psychosocial aspects of care (such as trauma informed care) and experience barriers to implementing trauma-informed care, in particular related to time and other resources available.

## 1. Methods

### 1.1. Design

The data used in this study were collected as part of a worldwide survey of emergency staff (participants in this study were included in the analysis of the worldwide survey). The study design and data collection procedures have been previously described (Alisic et al., 2016). The Human Research Ethics Committee of the Royal Children’s Hospital Melbourne granted ethics approval for this study (HREC 33,085).

### 1.2. Measure

We utilized four subscales (training experiences and preferences, knowledge of paediatric medical traumatic stress, barriers to implementing psychosocial care, attitude towards psychosocial care) of the Psychosocial Care Survey (described in Alisic et al., 2016) to assess: (1) the training status and preferences of emergency staff in LMICs; (2) emergency staff’s knowledge of paediatric medical traumatic stress; (3) perceived barriers to providing psychosocial care; and (4) emergency staff’s attitudes to providing psychosocial care. The measure was available in 12 language versions (English, French, Italian, Arabic, Spanish, Russian, Japanese, Cantonese, Mandarin, Hindi, Malay, Dutch). The training scale included eight items assessing participant’s prior training or education regarding psychosocial care for injured children, their desire to undertake training in this area and preferences for the format of training. The knowledge scale includes seven multiple choice questions (scored as ‘correct’ or ‘not correct’) that address ED staff knowledge of risk factors for the development of paediatric medical traumatic stress including characteristics of the child and family, characteristics of the injury, the child’s experience of the injury, behavioural presentations and the prevalence rate. The knowledge scale has strong internal consistency (Kuder Richardson 20 = .86) and one-month test-retest reliability (*r* = .75) (Kuder & Richardson, 1937; Pallant, 2007). The barriers scale included six items with a 3-point Likert scale, ranging from 1 = not a barrier to 3 = a significant barrier, with higher score indicating greater barriers to implementing psychosocial care. The barriers scale has acceptable internal consistency (Cronbach’s alpha = .75). The attitudes scale included 18 elements of trauma-informed psychosocial care, participants were asked to indicate if they viewed each aspect as part of their role. This scale had a high level of internal consistency (Cronbach’s alpha = .92). A positive attitude was operationalized as respondents viewing all aspects of psychosocial care as part of their role.

### 1.3. Data analysis

Data were collected via SurveyMonkey (www.surveymonkey.net) and analysis was conducted using SPSS version 21 (IBM, Armonk, NY, USA). Values of *p* < .05 were considered to be statistically significant. A total knowledge score was calculated as the total correct answers to the seven knowledge questions.

We employed descriptive statistics to describe respondent characteristics, knowledge of paediatric medical traumatic stress, attitudes to trauma-informed care, training status and preferences, and barriers to implementing trauma-informed care. A multiple regression analysis was performed with knowledge of paediatric medical traumatic stress as the dependent variable and the following as independent variables (years of experience, training in trauma-informed care, percentage of respondent’s patients who are children, country income). Due to the overwhelming majority of respondents being physicians we did not analyse the effect of profession on knowledge, attitudes, training preferences or barriers experienced. Missing data due to dropout was observed for 16% of participants, the remainder of the missing data was the result of skip logic.

### 1.4. Participants

We recruited respondents via the Pediatric Emergency Research Network (Klassen et al., 2010; PERN) and professional organizations throughout LMICs. In order to recruit as many respondents as possible from LMICs we utilized a snowball approach and asked respondents to forward the details of the survey to colleagues and other emergency medical professionals. Due to this approach we were unable to determine a response rate. Respondents eligible for inclusion in the analysis were emergency medical or nursing staff working in LMICs, as classified by the World Bank (World Bank, 2016).

Initial analysis of the data from LMIC respondents (*n *= 779) showed that a large number were from China (*n *= 463), which had the potential to bias the data. To address this, we selected a random sample of 42 cases (equal to the next largest sample from any one country) from China using a random number generator to be included in the analysis. Table 1 shows the distribution of respondents working in each country in the final sample.

**Table 1.** Distribution of respondents by country of work (*N* = 320).

*Country of work*	*n* (%)
**Low income countries**	
Congo, Democratic Republic of the (Zaire)	1 (0.3)
Ethiopia	2 (0.6)
Haiti	1 (0.3)
Liberia	2 (0.6)
Nepal	10 (3.1)
Sierra Leone	1 (0.3)
Somalia	1 (0.3)
Tanzania	1 (0.3)
Uganda	1 (0.3)
**Lower middle income countries**	
Angola	1 (0.3)
Bangladesh	1 (0.3)
Bolivia	1 (0.3)
Cameroon	2 (0.6)
Egypt	1 (0.3)
Georgia	1 (0.3)
Ghana	1 (0.3)
Guatemala	4 (1.3)
India	19 (5.9)
Kosovo	7 (2.2)
Laos	1 (0.3)
Lesotho	1 (0.3)
Libya	1 (0.3)
Mongolia	2 (0.6)
Nigeria	17 (5.3)
Pakistan	2 (0.6)
Papua New Guinea	2 (0.6)
Philippines	3 (0.9)
Sao Tome and Principe	1 (0.3)
Sri Lanka	3 (0.9)
Sudan	2 (0.6)
Zambia	4 (1.3)
**Upper middle income countries**	
Albania	13 (4.1)
Algeria	2 (0.6)
Argentina	42 (13.1)
Belize	1 (0.3)
Bosnia-Herzegovina	3 (0.9)
Botswana	5 (1.6)
Brazil	4 (1.3)
Bulgaria	2 (0.6)
China	42 (13.1)
Costa Rica	10 (3.1)
Cuba	1 (0.3)
Ecuador	2 (0.6)
Fiji	10 (3.1)
Hungary	2 (0.6)
Iran	12 (3.8)
Iraq	5 (1.6)
Lebanon	1 (0.3)
Malaysia	16 (5.0)
Marshall Islands	2 (0.6)
Mayotte	4 (1.3)
Mexico	3 (0.9)
Namibia	1 (0.3)
Romania	3 (0.9)
Saint Vincent and Grenadines	1 (0.3)
South Africa	28 (8.8)
Thailand	5 (1.6)
Turkey	3 (0.9)
Total	320 (100)

Note: Country of income classification as classified by the World Bank at the time of data collection, 2013 financial year (World Bank, 2016).

## 2. Results

The final sample consisted of 320 emergency staff from 58 LMICs. Table 2 shows the characteristics of the sample.

**Table 2.** Characteristics of survey respondents (*N* = 320).

Characteristic	
Age	40.2 (9.6)
*M* (*SD*)	
Gender, *n* (%)	135 (42.2)
Female	
Profession, *n* (%)	38 (11.9)
Nurse	282 (88.1)
Physician	
Location, *n* (%)	
Rural area	26 (8.1)
Suburban area	45 (14.0)
Urban area	249 (77.8)
Percentage of primary patients who are children, *n* (%)	
Less than 20%	103 (32.2)
20–40%	89 (27.8)
40–60%	21 (6.6)
60–80%	11 (3.4)
More than 80%	96 (30.0)
Regions within low/middle income countries, *n* (%)	
East Asia & Pacific	83 (25.9)
Europe & Central Asia	34 (10.6)
Latin America & Caribbean	70 (21.9)
Middle East & North Africa	22 (6.9)
South Asia	35 (10.9)
Sub-Saharan Africa	76 (23.8)
Years of experience in patient care	
*M* years (*SD*)	14.5 (9.6)

### 2.1. Training and education on psychosocial care for injured children

The overwhelming majority (91.4%) of respondents had not received any training or education in any type of psychosocial care for injured children. Additionally, 94.4% of respondents indicated they wanted this kind of training and education. Approximately one-third of respondents (37.8%) indicated that their first preference for training was via an online format, either by an interactive website (19.1%) or a website with written information (18.7%). Table 3 shows respondents training experience and preferences for training.

**Table 3.** Respondents training experience and preferences for training in paediatric medical traumatic stress and trauma-informed care.

Training status or preference	*n* (valid %)
Previous training in trauma-informed care, *n* (valid %)	
No training	244 (91.4)
Have had training	23 (8.6)
Further training in trauma-informed care, *n* (valid %)	
Want training	254 (94.4)
Do not want training	15 (5.6)
First preferences for mode of training *n* (valid %)	
A book on the topic	38 (14.2)
Group training in-person in one block of hours	26 (9.5)
Online: interactive website (e.g. webinar, video examples, quizzes)	51 (19.1)
Online: website and written information	50 (18.7)
Group training in-person spread over a number of weeks	51 (19.1)
Individual mentor sessions with an experienced clinician of my own profession	21 (7.9)
Individual mentor sessions with a mental health clinician	15 (5.6)

Note: *N* = 320, valid % indicates percentage of respondents excluding missing data.

### 2.2. Knowledge of paediatric medical traumatic stress

On average, respondents correctly answered 2.2 (*SD* = 1.6) of the seven knowledge questions with no respondents answering all knowledge questions correctly. The level of knowledge amongst respondents varied across the areas of knowledge of paediatric medical traumatic stress examined (see Table 4). Relative strengths included recognizing that the development of paediatric medical traumatic stress is associated with the child and family’s subjective appraisal of both pain (50.9% correct) and life threat (46.9% correct), rather than objective injury severity (41.9% correct). In addition, respondents demonstrated an awareness that the child, parents and siblings are all at risk of paediatric medical traumatic stress following paediatric injury (41.6% correct). However, while these are highlighted as relative strengths it is important to note that less than half of all respondents were aware of these risk factors.

**Table 4.** Descriptive analysis of LMIC emergency staff knowledge of paediatric medical traumatic stress, correct answers.

Knowledge items	Answered correctly, *n* (%)
All levels of injury severities are at risk for traumatic stress	134 (41.9)
All age groups are at risk for traumatic stress	67 (20.9)
Child/parents/siblings are at risk	133 (41.6)
Various behaviours (e.g. calm, frantic) can indicate risk	37 (11.6)
Subjective life threat is risk factor	150 (46.9)
Pain is a risk factor	163 (50.9)
> 50% of children report stress symptoms in 1st month post-injury	24 (7.5)

Note: *N* = 320.

Weaknesses in knowledge of paediatric medical traumatic stress included the prevalence of children who experience symptoms in the first month following the injury (7.5% correct) and the insight that various behaviours in the immediate aftermath can indicate a child or family is at risk of developing paediatric medical traumatic stress (11.6% correct). In addition, relatively few respondents could correctly recognize that all age groups are at risk (20.9% correct) and that paediatric medical traumatic stress can affect toddlers (30.3% correct).

### 2.3. Predictors of knowledge

The combination of all variables significantly predicted knowledge of paediatric medical traumatic stress and explained 9% of the variance in the model (*R*^2^ = .09, *F*(5, 261) = 5.02, *p* < .001; see Table 5). The variables ‘training in psychosocial care’ (*p* < .001) and ‘country income’ (*p* = .006) uniquely predicted knowledge scores (see Table 5), indicating that higher levels of knowledge were associated with receiving training in psychosocial care and being from a higher income country. Years of experience (*p* = .46), confidence in psychosocial care (*p* = .30) and the percentage of patients who are children (*p* = .16) were not unique significant predictors of emergency medical staff knowledge of paediatric medical traumatic stress.

**Table 5.** Multiple regression analysis of emergency staff’s knowledge of paediatric medical traumatic stress.

Variable	*B*	SEB	β
Years of experience in patient care	0.01	0.01	0.05
Training in trauma-informed care (no training, had training)	1.01	0.33	0.19**
Percentage of primary patients who are children	0.08	0.06	0.09
Confidence in psychosocial care	0.01	0.01	0.06
Country income according to world bank categories	0.17	0.06	0.17*

Note: *n* = 267; * = *p *< .05; ** = *p <* .001; *F*(5,261) = 5.02; *p* < .001; *R*^2^ = .09; *B* = unstandardized regression coefficient; SEB = Standard error of the coefficient; β = standardized coefficient; ‘No training’ was codded ‘0ʹ; ‘Have had training’ was coded ‘1’.

### 2.4. Barriers experienced to providing trauma-informed care

Table 6 shows the barriers that respondents indicated they experience to implementing trauma-informed care. The most frequently endorsed barrier was a lack of training with 87.5% of respondents indicating that this affected their ability to implement trauma-informed or psychosocial care. However, all barriers were endorsed by the vast majority of respondents.

**Table 6.** Barriers to implementing trauma-informed care experience by emergency staff.

Barriers to implementing psychosocial care	Staff who considered this a barrier *n* (valid %)
Time constraints	233 (82.9)
Lack of training	246 (87.6)
Confusing evidence on what to do	222 (79.0)
Worry about further upsetting children and families	191 (68.0)
A lack of dedicated space to provide psychosocial care	235 (83.6)
Lack of support from supervisors or others in the health care system	225 (80.1)

Note: *N* = 320, valid % indicates percentage of respondents excluding missing data.

### 2.5. Attitudes towards trauma-informed care

Respondents had positive attitudes to psychosocial care, with 82.3% indicating that all aspects of psychosocial care were part of their role.

## 3. Discussion

Overall the results of this study indicated that emergency staff working in LMICs have gaps in knowledge of paediatric medical traumatic stress and express a need and want for training in psychosocial care for injured children (i.e. trauma-informed care). Consistent with observations from past research, greater knowledge was associated with higher country income (Alisic et al., 2016; Chandran et al., 2010; Fodor et al., 2014; Schnyder et al., 2016). LMIC emergency staff had somewhat greater gaps in knowledge than their high-income country counterparts and in particular were less likely to identify that toddlers can be at risk of developing paediatric medical traumatic stress and recognize behaviours that indicate risk (Alisic et al., 2016; Hoysted et al., 2017). Conversely, LMIC emergency staff demonstrated greater awareness of the impact of pain on the risk of developing paediatric medical traumatic stress (Alisic et al., 2016; Hoysted et al., 2017). In the current analysis, LMIC emergency staff demonstrated a moderate level of knowledge of some risk factors for the development of paediatric medical traumatic stress. Despite this, overall rates of knowledge amongst respondents are low and indicate substantial room for improvement. The overall limited knowledge (including the prevalence of paediatric medical traumatic stress, behaviours that indicate risk, and age groups at risk of paediatric medical traumatic stress) suggests that without training respondents would be unlikely to be able to identify children and families at risk of developing paediatric medical traumatic stress. This is likely to be a barrier to the use of simple low-cost prevention measures such as information provision or stepped care appro-aches (Kassam-Adams, 2014; Kenardy, Thompson, Le Brocque, & Olsson, 2008). As such, training and education is required to improve awareness of paediatric medical traumatic stress in conjunction with training in trauma-informed care.

Emergency staff in LMICs reported experiencing significant barriers to providing trauma-informed care. Consistent with previous research conducted amongst both emergency staff and paediatricians in high-income countries, a lack of training and confusing evidence on what to do to assist children and families were identified as barriers to the provision of trauma-informed care (Banh et al., 2008; Horowitz et al., 2001). The experience of systemic barriers to the implementation of trauma-informed care (including a lack of support from supervisors and the system as a whole in doing so) are consistent with past research which has identified poor governance as a barrier to the integration of mental health care in LMICs (Petersen et al., 2017). Lack of referral resources for patients identified as being at risk compounds the problem as mental health resources, particularly those tailored to child and adolescent patients, are scarce in many LMICs (Kakuma et al., 2011; Kieling et al., 2011). In addition to improving knowledge, reducing systemic barriers is an important step in creating trauma-informed health care systems, which requires organizations to be committed to addressing the impact of trauma on children and families (Kassam-Adams, 2014; Ko et al., 2008; Marsac et al., 2015; Petersen et al., 2017). Further, as time constraints were frequently identified as a barrier to providing trauma-informed care, training programmes that are developed for staff in these regions should aim to be brief and time efficient. It is important to note that medical and nursing staff would not be expected to replace mental health workers but rather provide routine medical care in a way that acknowledges the impact of trauma and minimizes distress, thus altering the subjective experience of the event and the immediate aftermath (Kazak et al., 2006; Marsac et al., 2014; Price et al., 2015). Several preventative approaches including the DEF protocol (Stuber, Schneider, Kassam-Adams, Kazak, & Saxe, 2006) which provide evidence based guidelines on implementing trauma-informed care and information provision (Kenardy et al., 2008) are designed to be incorporated into routine care and add little burden on time (in the Medical Traumatic Stress Toolkit: Kassam-Adams, 2014; Kenardy et al., 2008; Ko et al., 2008; Marsac et al., 2015).

Despite experiencing significant barriers to providing trauma-informed care, respondents were found to hold favourable attitudes to trauma-informed care with the overwhelming majority considering psychosocial care to be part of their job. This is an important finding in conjunction with the finding that the overwhelming majority of respondents expressed a desire for training in this area, as positive attitudes have been identified as a key determinant in the successful implementation of evidence based interventions (Pentland et al., 2011; Varnell, Haas, Duke, & Hudson, 2008), such as trauma-informed care.

Our findings indicate that training in this area would be useful for emergency staff of all levels of experience, although the greatest benefit might be for those working in comparatively lower-income countries. In addition to training, both emergency staff and children and families should be provided with user-friendly screening and education tools (Kazak, 2006; Price et al., 2015). Whilst we acknowledge that the need for medical training must take priority (Obermeyer et al., 2015), we would argue that training and education on paediatric medical traumatic stress and trauma-informed care would be valuable considering the high prevalence of trauma. Unlike resource-intensive medical training, basic education on paediatric medical traumatic stress and trauma-informed care can be developed at a low cost and widely distributed online among LMIC emergency staff without placing substantial burden on time. Further, training in this format would meet the preferences of emergency staff in this region, as identified in this study, and may contribute to enhanced services provided by emergency staff. For this approach to be successful consideration would have to be given to the online format, for instance ensuring access would be feasible in areas with limited or variable bandwidth (such as content that can be downloaded on to all device types and saved when sufficient bandwidth is available). This format would meet the requirements identified in the literature for the development of interventions that are feasible in the LMIC context, as trauma-informed care can be incorporated into existing care models utilizing existing human resources (Kassam-Adams, 2014; Patel et al., 2007, 2011; van Ginneken et al., 2013). Examples of existing training programmes that could be implemented in LMICs include Psychological First Aid based programmes and guides (World Health Organization War Trauma Foundation and World Vision International, 2011).

While such training should be adapted to the local context there is evidence for the applicability of interventions, developed in Western systems, for posttraumatic stress and related disorders being successfully implemented in culturally diverse LMIC settings following disasters (Kahana, Feeny, Youngstrom, & Drotar, 2006; Schnyder et al., 2016). Patel et al. (2007) describes the process of cultural adaptation of mental health interventions for LMICs through consultation with key stakeholders, piloting of the revised programme and an understanding of the feasibility and sustainability of the intervention. Importantly the findings of this process of cultural adaptation demonstrated that the broad components of the mental health interventions examined were cross-culturally acceptable however modifications were needed to ensure the feasibility and acceptability. As such, the development of training and interventions for staff in this region should include consultation with key stakeholders to ensure they are culturally appropriate prior to their implementation. A recent study has demonstrated promising results with a brief, low-cost training programme for ED staff, which could be adapted for use in LMICs (Hoysted, Jobson, & Alisic, In press).

This study examined emergency staff’s knowledge and perspectives; however, conclusions cannot be made regarding the current level of skills that LMIC emergency staff have or the frequency at which they provide elements of trauma-informed care. Furthermore, there is substantial variation of country income, access to resources and trauma care systems within LMICs: this paper provides a broad overview of knowledge and training across countries. In addition, we used online self-report measures and as such have excluded clinicians without Internet access. Although this gives novel insights in an area of importance, we may have had greater participation by those with a preference for accessing online materials. Participation in this study was both voluntary and anonymous and the recruitment strategy involved a snowball approach to ensure the survey was distributed to as many respondents in LMICs as possible. Thus, the nature of this recruitment approach allows the possibility of a self-selection bias where participants who volunteered may have been more likely to have an interest in mental health or trauma-informed care. In addition, given that the majority of countries were represented by less than five respondents, the sample may represent a select group of emergency staff.

## 4. Conclusion

Emergency staff in LMICs were found to have relatively limited knowledge of paediatric medical traumatic stress. A higher level of knowledge of posttraumatic stress in injured children was associated with having had training in psychosocial care and working in a higher income country within LMICs. There is a need and desire for training in psychosocial care for injured children care amongst emergency staff in LMIC. Providing training and education in paediatric medical traumatic stress and trauma-informed care in this region may improve outcomes for children with acute injuries. Encouragingly, despite experiencing significant barriers to providing trauma-informed care, emergency staff in LMICs were found to hold favourable attitudes to providing trauma-informed care. Widespread training provided to emergency staff in LMICs, trauma-informed care for injured children could provide a preventative intervention that can be universally accessed by all receiving emergency medical treatment.
